# Chronic idiopathic systemic capillary leak syndrome: a case report

**DOI:** 10.1186/s13223-019-0347-0

**Published:** 2019-05-28

**Authors:** Ahmed M. Alkhunaizi, Abdullah H. Kabbani, Mohamed A. ElTigani

**Affiliations:** 1 Nephrology Section, Internal Medicine Services Division, Johns Hopkins Aramco Healthcare, Saudi Aramco, Box 10955, Dhahran, 31311 Saudi Arabia; 2 General Internal Medicine, Johns Hopkins Aramco Healthcare, Dhahran, Saudi Arabia; 3 Faculty of Medicine, Imam Abdulrahman Bin Faisal University, Dammam, Saudi Arabia

**Keywords:** Capillary leak syndrome, Clarkson disease

## Abstract

**Background:**

Idiopathic systemic capillary leak syndrome (ISCLS), is a rare disorder characterized by recurrent attacks of varying severity of hypovolemic shock and generalized edema in association with hemoconcentration and hypoalbuminemia in the absence of albuminuria. The chronic form of ISCLS is extremely rare with only a few cases reported in the literature.

**Case presentation:**

Here we report the case of a young woman who developed chronic ISCLS characterized by massive, generalized and persistent edema. The work up confirmed the presence of monoclonal gammopathy. She was treated with several agents with no response.

**Conclusion:**

Chronic ISCLS is a very rare disease of unknown etiology and results in devastating complications. The medical community should be aware of this disease with the hope that targeted therapy will become available in the future.

## Case

**Fig. 1:**
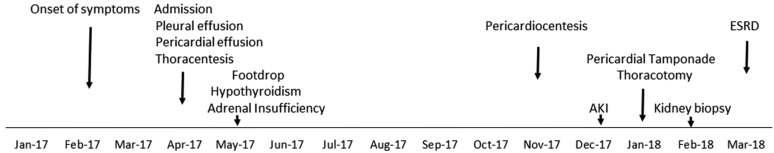
Timeline of clinical events. *AKI* acute kidney injury, *ESRD* end stage renal disease

A 23-year-old female patient who is known to have type 1 diabetes mellitus since the age of 12 years. Her blood sugar had been poorly controlled. She was transferred to our hospital from another institution in April, 2017 for evaluation of anasarca. Two months prior to her admission she started developing generalized body swelling with no associated arthritis, arthralgia or hair loss. There was no family history of similar illness. Her weight was 140 kg. Her weight prior to her illness was around 70 kg. Her exam was significant for massive anasarca involving all extremities, abdominal wall and back. She developed bilateral pleural effusions and pericardial effusion with tamponade. She later on developed bilateral foot drop, adrenal insufficiency, hypothyroidism, acute kidney injury and end stage renal disease (ESRD) (Fig. 1).

## Laboratory tests

Admission hemoglobin was 13.7 (12.0–16.0) gm/dL. The white blood cell count with differential and platelet counts were normal. Renal function and liver function were normal. Urinalysis showed no proteinuria. Random urine creatinine was 199 mg/dL and urinary sodium was 8 mmol/L. B-type natriuretic peptide: 18. Serum albumin was 3.1 (3.4–4.7) g/dL. Thyroid stimulating hormone was 5.18 (0.35–4.94) μIU/mL, free T4: 0.93 (0.68–2.53) ng/dL and free T3: 1.62 (1.71–3.71) pg/mL. The chest X-ray showed bilateral pleural effusions. The echocardiogram showed normal ejection fraction greater than 55% and a large pericardial effusion with impending tamponade. The pleural fluid analysis showed: LDH: 65 U/L, total protein: 2.7 g/dL (simultaneous serum sample: protein 5.2 (6.3–7.9) g/dL, LDH: 133 (120–246) U/L. The anti-nuclear antibodies were positive at 1:80 with speckled pattern. The anti-double-stranded deoxyribonucleic acid (DNA) was negative. The antibodies to extractable nuclear antigens were all negative. C-reactive protein was 0.8, and erythrocytes sedimentation rate was 8 mm/hr. C3:91 (9–180), C4:23 (10–40) mg/dL and total serum complements (CH50) were 45 (30–75) U/mL. Hepatitis B, C and HIV serologies were negative. Tissue glutaminase IgA antibodies were < 1.2 U/mL (normal < 4.0 U/mL). Serum protein electrophoresis showed: total protein 4.9 (6.3–7.9) g/dL, albumin: 2.2 (3.4–4.7) g/dL, alpha-1 globulin: 0.2 (0.1–0.3) g/dL, alpha-2 globulin: 0.9 (0.6–1.0) g/dL, beta-globulin: 0.6 (0.7–1.2) g/dL, gamma-globulin: 1.0 (0.6–1.6) g/dL, albumin/globulin ratio: 0.84. The serum immunofixation showed small monoclonal IgG kappa within the gamma fraction. Kappa and lambda free light chains were 5.54 and 1.80 mg/dL respectively. The Kappa/lambda ratio was 3.08. Total IgG: 936 (767–1590) mg/dL, the IgG subclasses: IgG1: 704 (341–894) mg/dL, IgG2: 66 (171–632) mg/dL, IgG3: 20.9 (18.4–106) mg/dL, IgG4: < 0.3 (2.4–121) mg/dL. C1 esterase inhibitor level was 33 (19–37) mg/dL. Total vitamin D was 5.0 (25–80) ng/mL. Abdominal fat biopsy was negative for amyloid deposition by Congo red stain.

## Hospital course

She required thoracentesis repeatedly to treat the persistent pleural effusion. She also required pericardiocentesis. She was tried on multiple medications that have been shown in earlier case reports to be effective in treating capillary leak syndrome including: high dose systemic corticosteroids, several doses of intravenous immunoglobulins (IVIG) followed by monthly administration for 6 months (2 gm/kg body weight), intravenous Theophylline, Terbutaline, Bevacizumab (two doses 2 weeks apart), intravenous methylene blue, and Thalidomide for 4 months. She did not respond to any of these modalities (Table 1).

**Table 1:** Therapeutic agents used during the course of illness

Agent	Dose	Duration	Response
Methyl-prednisone	500 mg/day	Several doses	No
IVIG	2 gm/kg	Several doses followed by monthly for 6 months	No
Intravenous theophylline	5 mg/kg followed by 0.4 mg/kg/h	2 days	No
Terbutaline	5 mg three times/day	14 days	No
Bevacizumab	5 mg/kg	2 doses 2 weeks apart	No
Intravenous methylene blue	2 mg/kg	One dose	No
Thalidomide	200 mg daily	4 months	No

*IVIG* intravenous immunoglobulins

She had developed recurrent episodes of sepsis related to central line infection and pneumonia and required transfer to the intensive care unit on multiple occasions. She also developed pericardial tamponade and required thoracotomy, pericardial drainage and creation of a large pericardial window. As a result of the septic episodes and the cardiac procedure, she developed acute kidney injury and was started on continuous renal replacement therapy and later was switched to intermittent hemodialysis. After 6 weeks on dialysis, a kidney biopsy was performed and showed acute tubular necrosis with regenerating renal tubules. She however did not recover and became dialysis dependent requiring daily dialysis in an attempt to control the edema. After 24 months of continuous hospitalization with frequent transfers to the step down unit and intensive care unit, she still has massive edema mainly in the lower extremities requiring daily dialysis, is bedbound, and has bilateral foot drop. She has suffered from immobilization hypercalcemia, bone fractures and decubitus ulcerations.

## Discussion

The patient represents a rare case of severe and chronic ISCLS where she developed massive and persistent generalized edema in addition to bilateral pleural effusion and pericardial effusion. The initial laboratory values were normal except for the presence of monoclonal gammopathy.

The classic systemic capillary leak syndrome is a rare disease of reversible plasma extravasation and vascular collapse accompanied by hemoconcentration and hypoalbuminemia [1, 2]. The first description of the syndrome was by Clarkson et al. who described a case of a 32-year-old woman who experienced “a strange cyclical illness in which she intermittently had a sudden massive movement of plasma from her vascular bed [3]. Typically, patients experience the development of massive edema and shock after a nonspecific prodrome of weakness, fatigue, and myalgias. They may develop ischemia-induced organ failure, rhabdomyolysis, compartment syndromes, and venous thromboembolism. These manifestations may rapidly resolve and patients may experience recurrent and intermittent attacks with spontaneous recovery. Less than 500 cases have been reported in the literature since the initial description by Clarkson et al. [4]. On the other hand the chronic form of ISCLS is extremely rare with only a few cases reported in the literature [5–8]. Unlike the classic form of ISCLS, chronic ISCLS is characterized by persistent edema with no signs of recovery and may be responsive to treatment with systemic steroids and IVIG [5, 7]. Similar to our case, monoclonal gammopathy is classically found in both forms of ISCLS. ISCLS is a life-threatening disorder that carries a high mortality rate. The pathogenesis is poorly understood and is believed to be a manifestation of transient endothelial dysfunction due to endothelial contraction, apoptosis, injury, or a combination of these factors [1]. Exaggerated microvascular endothelial responses to surges of otherwise routinely encountered inflammatory mediators have been also suggested [4]. Certain cytokines were found to be elevated in acute ISCLS sera compared to baseline or sera from healthy controls, including CXCL10, CCL2, IL-1β, IL-6, IL-8, IL-12 and tumor necrosis factor α (TNFα) [9].

In severe forms, patients may develop persistent and chronic edema with no sign of recovery. Our patient represents this type of chronic ISCLS where she developed severe and persistent generalized edema and showed no response to any of the modalities that had been previously tried. She also developed hypothyroidism and adrenal insufficiency which may be related to extravasational loss of hormones and their serum binding proteins [10].

The diagnosis of ISCLS is made clinically after excluding conditions that may have a similar presentation such as sepsis, anaphylaxis and angioedema. There is no established treatment for ISCLS and management is mainly supportive directed towards correction of intravascular volume depletion, maintaining organ perfusion, and avoiding severe metabolic acidosis. Crystalloids and or protein containing solutions may be used to maintain hemodynamic stability. Caution must be exercised as excessive fluid administration may lead to worsening edema and development of compartment syndrome. Different modalities have been tried to treat this devastating illness with some success in case reports and case series including vascular endothelial growth factor (VEGF), TNFα, Theophylline, IVIG, Terbutaline and Thalidomide [11–20].

In conclusion, we have described a case of severe chronic ISCLS that has not responded to any modality of treatment. The patient ended up with devastating complications. The medical community should be aware of this disease with the hope that targeted therapy will become available in the future.

## Data Availability

Not applicable.

## Abbreviations

ISCLS: idiopathic systemic capillary leak syndrome
DNA: deoxyribonucleic acid
IVIG: intravenous immunoglobulins
VEGF: vascular endothelial growth factor
IL: interleukin
